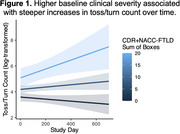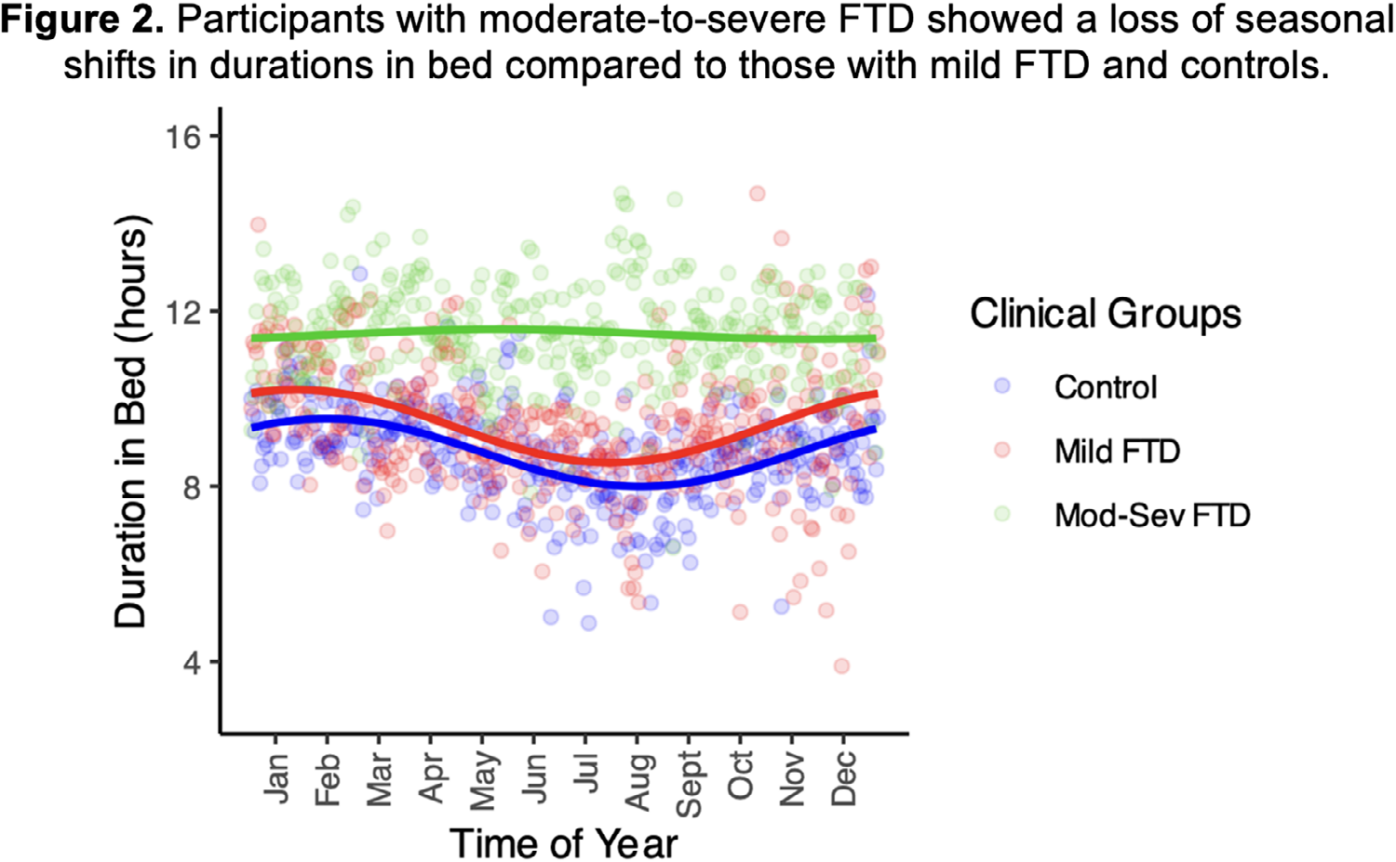# Continuous at‐home monitoring of sleep behavior in frontotemporal lobar degeneration

**DOI:** 10.1002/alz70857_104745

**Published:** 2025-12-25

**Authors:** Emily W. Paolillo, Amy B. Wise, Sreya Dhanam, Anjali Sadarangani, Wan‐Tai Michael Au‐Yeung, Zachary T Beattie, Jeffrey A Kaye, Kaitlin B Casaletto, Rowan Saloner, Mario F. Mendez, William W. Seeley, Maria Luisa Gorno Tempini, Adam L. Boxer, Howard J. Rosen, Christine M Walsh, Adam M. Staffaroni

**Affiliations:** ^1^ Memory and Aging Center, UCSF Weill Institute for Neurosciences, University of California, San Francisco, San Francisco, CA, USA; ^2^ Memory and Aging Center, UCSF Weill Institute for Neurosciences, University of California San Francisco, San Francisco, CA, USA; ^3^ Oregon Health & Science University, Portland, OR, USA; ^4^ University of California, Los Angeles, Los Angeles, CA, USA; ^5^ Department of Neurology, Memory and Aging Center, University of California San Francisco, San Francisco, CA, USA; ^6^ Memory and Aging Center, Department of Neurology, Weill Institute for Neurosciences, University of California, San Francisco, San Francisco, CA, USA

## Abstract

**Background:**

Sleep and circadian disturbances are common yet understudied in frontotemporal dementia (FTD). Advances in non‐invasive sleep monitoring technology capture real‐time objective sleep behaviors in naturalistic settings. We examined baseline and longitudinal nighttime bed behaviors using an under‐mattress sensor in adults with FTD.

**Method:**

Participants included 16 adults with FTD syndromes and 12 study partner controls recruited from UCSF and UCLA (mean age = 67.4±7.2; 54% male). An Emfit^TM^ flexible movement/pressure sensor was placed under each participant's mattress at thoracic level to monitor bed behaviors for up to two years (mean = 349.6±182.6 days monitored; range=144‐700 days). Metrics were derived from each session in bed: duration in bed, bed‐entry/exit clock times, number of tosses/turns, and number of bed‐exits. Pearson correlations examined relationships between bed behaviors and clinical severity (CDR+NACC FTLD sum of boxes) at baseline (defined as first 30 days). Linear mixed‐effects regressions examined longitudinal associations between baseline clinical severity and bed behaviors over time. Finally, to examine differential seasonal shifts in nightly bed behaviors, Fourier basis functions modeled nightly duration in bed over a full calendar year separately by clinical severity groups (controls vs. mild FTD vs. moderate‐to‐severe FTD).

**Result:**

At baseline, greater clinical severity associated with higher average duration in bed (*r* = 0.51, *p* = 0.006), average toss/turn count (*r* = 0.53, *p* = 0.005), and average bed exit count (*r* = 0.40, *p* = 0.041), but not variability in duration or bed‐entry/exit clock times across days (*p*s>0.05). Longitudinally, person‐specific linear trajectories of bed behaviors revealed that higher baseline clinical severity associated with steeper increases in tosses/turns over time (β=0.16, *p* = 0.026; Figure 1). Those with more severe FTD also had an attenuated behavioral response to seasonal daylight shifts (Figure 2). That is, unlike those with mild FTD and controls, those with moderate‐to‐severe FTD did not have altered durations in bed relative to seasonal daylight hours (β=0.23, *p* <0.001).

**Conclusion:**

Findings demonstrate the feasibility of long‐term, non‐invasive nighttime behavior monitoring using bed sensors, highlighting their utility to monitor disease progression in FTD. Further research is needed to explore the underlying mechanisms of sleep disturbances in FTD and develop targeted interventions to improve sleep and subsequent quality of life for individuals with FTD.